# Safety, efficacy and delivery of isometric resistance training as an adjunct therapy for blood pressure control: a modified Delphi study

**DOI:** 10.1038/s41440-021-00839-3

**Published:** 2022-01-12

**Authors:** Biggie Baffour-Awuah, Melissa J. Pearson, Neil A. Smart, Gudrun Dieberg

**Affiliations:** 1 Clinical Exercise Physiology, School of Science and Technology, Faculty of Science, Agriculture, Business and Law, University of New England, Armidale, NSW 2351 Australia; 2 Biomedical Sciences, School of Science and Technology, Faculty of Science, Agriculture, Business and Law, University of New England, Armidale, NSW 2351 Australia

**Keywords:** Blood pressure, Delphi, Isometric resistance training

## Abstract

Uncontrolled hypertension remains the major risk factor for cardiovascular disease. Isometric resistance training (IRT) has been shown to be a useful nonpharmacological therapy for reducing blood pressure (BP); however, some exercise physiologists and other health professionals are uncertain of the efficacy and safety of IRT. Experts’ consensus was sought in light of the current variability of IRT use as an adjunct treatment for hypertension. An expert consensus-building analysis (Delphi study) was conducted on items relevant to the safety, efficacy and delivery of IRT. The study consisted of 3 phases: (1) identification of items and expert participants for inclusion; (2) a two-round modified Delphi exercise involving expert panelists to build consensus; and (3) a study team consensus meeting for a final item review. A list of 50 items was generated, and 42 international experts were invited to join the Delphi panel. Thirteen and 10 experts completed Delphi Rounds 1 and 2, respectively, reaching consensus on 26 items in Round 1 and 10 items in Round 2. The study team consensus meeting conducted a final item review and considered the remaining 14 items for the content list. A final list of 43 items regarding IRT reached expert consensus: 7/10 items on safety, 11/11 items on efficacy, 10/12 items on programming, 8/10 items on delivery, and 7/7 on the mechanism of action. This study highlights that while experts reached a consensus that IRT is efficacious as an antihypertensive therapy, some still have safety concerns, and there is also ongoing conjecture regarding optimal delivery.

## Introduction

Chronic disease is the leading cause of death and disability globally, with a disease burden accounting for 71% (41 million) of all deaths each year. Chronic disease occurs subsequent to genetic, physiological, environmental and behavioral factors and often has long-lasting consequences [1]. The majority of deaths (85%) between the ages of 30 and 69 years are premature, with cardiovascular diseases (CVDs) accounting for 15% (~18 million) of the total [1, 2]. In an attempt to reduce the burden of CVDs, the WHO supports the implementation of cost-effective interventions (both population-wide and individual) for the prevention and control of CVDs [3]. Hypertension remains the major risk factor for CVDs and many other medical conditions, causing sequelae that contribute to many chronic diseases. It is a major cause of premature death, with ~1.13 billion adults having hypertension worldwide. In two-thirds of affected people, hypertension is uncontrolled [4] and may lead to organ damage (affecting the heart, kidneys, brain, etc.) [5]. A 25% reduction in the prevalence of hypertension by 2025 (with 2010 as the baseline year) was established as one of the targets in the Global Action Plan for the Prevention and Control of Noncommunicable Diseases (2013–2020), which directly focuses on preventing and controlling CVDs [3]. The prevalence of hypertension increases significantly with age [6] despite efforts to control it. Suitable pharmacological intervention is vital in the management and treatment of hypertension; however, less than half of those treated worldwide experience effective blood pressure control [7]. A recent review showed that intensively treating BP lowers the relative risk of major cardiovascular events (26%) and all-cause mortality (18%); however, this often involves the use of multiple medications that have unavoidable additional side effects, some of which are secondary to hypotension [8]. This underscores the significance of delivery gaps and calls for alternative/adjunct antihypertensive therapies.

Population-wide interventions focus on lifestyle modifications; for example, exercise has a significant effect on BP in addition to other health benefits [9]. However, these interventions require long-term commitment for effective results, and poor adherence remains a major drawback [10–12]. Therefore, it is worth considering the cost and time efficiency of exercise interventions to increase compliance, as most patients will not adhere to this lifestyle modification [13]. Both dynamic resistance training (DRT) and aerobic exercise training (AET) are recommended for their antihypertensive effects, even though a study by Schroeder et al. [14] and a recent meta-analysis [15] suggest that combined training may provide more comprehensive CVD benefits than isolated aerobic or resistance training. Nevertheless, the exercise modality of AET is preferred over DRT, which is a recommended supplement, according to exercise guidelines for managing hypertension [16–28]. Participation in and adherence to AET remain low, most likely due to the time and cost [29, 30] involved in accessing a gymnasium or equipment (to accommodate orthopedic limitations) and the professional supervision that may be required for some high-risk populations [9].

Numerous randomized controlled trials have demonstrated the antihypertensive benefits of isometric resistance training (IRT) conducted in healthy individuals [31–36] and chronic disease patients [37–40]. Current meta-analyses [41–47] support the status of IRT as an emerging alternative antihypertensive therapy. Isometric resistance training is the execution of a static, sustained muscle contraction against an immovable source of resistance with little or no change in the length of the muscle group(s) involved [9]. Isometric resistance training produces a much lower rate-pressure product (RPP) index than either AET or DRT [48, 49]. The rate-pressure product, defined as the product of systolic blood pressure and heart rate, is used as a measure of myocardial oxygen consumption [50]. Some exercise experts as well as other health care specialists who utilize exercise as a treatment modality remain unconvinced as to the safety of IRT despite the existence of some guidelines that recommend IRT as an adjunct therapy to lower BP [16, 51]. The Canadian guidelines [19], for example, suggest the use of resistance or weight training exercise (such as free weight lifting, fixed weight lifting, or handgrip exercise) for pre-hypertensive or stage 1 hypertensive individuals, as this does not adversely influence BP. Similarly, a recently updated Exercise & Sport Science Australia (ESSA) position statement on exercise and hypertension recommends IRT as an adjunct antihypertensive therapy [52]. This is supported by a recent meta-analysis that shows no significant increase in risk when IRT is performed [53]. However, some guidelines do not mention IRT [17, 23, 26, 54, 55] or do not recommend it [24, 25] as a result of the perceived acute increase in BP during exercise. Hansen et al. [56], in their consensus statement from the Exercise Prescription in Everyday Practice and Rehabilitative Training (EXPERT) working group for exercise prescription in patients with different combinations of CVD risk factors, identified IRT as an adjunct tool to lower blood pressure [56]. They suggested that the paucity of data warranted further research (level of evidence: 2 + ; grade of recommendation: C) according to the grading recommendations in evidence-based guidelines [57] for exercise prescription. More importantly, an official document of the American College of Sports Medicine, recently published by Pescatello et al. [58], suggested that there is no conclusive evidence for the antihypertensive benefit of IRT, basing this statement solely on a previous meta-analysis [41]. There are more recent and robust meta-analyses [42, 44] as well as several recent empirical studies [45, 46, 53, 59, 60] that demonstrate the safety and effectiveness of IRT in managing hypertension. Similarly, recent work by Fu et al. [61] identified IRT as an effective antihypertensive therapy. These authors performed a network meta-analysis to synthesize direct and indirect evidence from studies that compared multiple interventions to lower BP (120 studies, including 22 with nonpharmacological interventions) [61]. Despite overwhelming evidence from RCTs, systematic reviews and meta-analyses as well as recommendations in international guidelines, there is ongoing conjecture about IRT. In light of this situation, a modified Delphi method was employed to seek expert consensus building with the aim of identifying the relevant issues worth considering for the safety, efficacy, and delivery of IRT as an adjunct therapy in the management of hypertension.

## Methods

The Delphi technique is commonly used in health care research and seeks opinions from a group of experts to assess the level of agreement and resolve disagreement on an important issue [62, 63]. In this study, a modified Delphi technique was used to build consensus [64] on a set of items generated through an extensive literature review. This project was approved by the Human Research Ethics Committee of the University of New England (Approval No. HE20-180).

Steered by the study team’s (BB, MP, GD and NS) evaluation of the literature on IRT research and current guidelines for the application of IRT, consensus building was conducted in three phases:

1. Identification of items regarding the safety, efficacy and delivery of IRT and expert participants for inclusion (August–October 2020);
2. A 2-round modified Delphi study conducted to build consensus (October 2020–January 2021); and
3. A consensus group meeting among the study team to finalize items (February 2021).

### Phase 1: Identification of items and expert participants for inclusion

After a careful review of the available literature investigating IRT and with reference to systematic reviews and meta-analyses [9, 41–44, 46, 59–61, 65–69], the study team reached an agreement on the inclusion/exclusion of potential items in relation to the antihypertensive effects of IRT. A set of statements was developed as items for the current study. These items were categorized under five subheadings: safety, efficacy, programming, delivery and mechanism of action of IRT. Some items negating previous statements were included to validate participants’ responses. A first draft of these items served as a pilot test for comments and suggestions before the questionnaire was finalized using the online survey software Qualtrics (Qualtrics, Provo, UT, USA).

Once the questionnaire was finalized, the experts were selected; an “expert” was defined as an individual involved in IRT research and/or the use of exercise intervention as an adjunct treatment method. The panelists were identified and selected based on their area of expertize and specified inclusion criteria (see below) [70]; these individuals included (1) exercise professionals working in a clinical or academic setting and (2) health care professionals with experience in the use of exercise as a treatment modality or experience in developing exercise guidelines.

Thus, the sampling technique was purposive and based on selected criteria [69]. Since IRT is an emerging area, the number of potential expert panelists worldwide is relatively low; therefore, panelists were recruited with no geographic limitations. Potential expert panelists were identified at tertiary education institutions, research institutes, and health care providers through IRT networks, internet searches and authorship of relevant publications. Their contact addresses (email) were obtained from their staff profiles on their organizations’ websites or from published studies.

The inclusion criteria for potential expert panelists were as follows:

1. Academics with at least one peer reviewed publication in isometric exercise research.
2. Exercise or healthcare professionals who
  - are specialists using exercise in the management of chronic diseases (especially cardiovascular diseases), and
  - have a relevant postgraduate qualification or at least 3 years’ experience in an exercise and sports or musculoskeletal setting.
3. Currently employed as an academic or professional in clinical practice.
4. At least 18 years old and proficient in English.

Potential expert panelists were included if they met criteria 1 and/or 2, plus mandatory criteria 3 and 4. Potential panelists were excluded if they were academics (criterion 1) with no IRT research publications.

### Phase 2: Modified Delphi study to build a consensus

The second phase of the study involved the distribution of invitation emails to the identified experts for inclusion as panelists. Qualtrics was used to communicate with experts/panelists, obtain their consent to participate, and administer the questionnaires [71]. Invited experts who consented to participate were given access to the survey to review each generated item and rate their agreement with it. Expert panelists were asked to complete two consecutive rounds of the Delphi process over 10 weeks and remained anonymous to the other panel members. During this period, a reminder email was sent a week before the end of each round, addressed only to panelists who had not yet responded. Panelists’ feedback and suggestions in Round 1 were incorporated into the Round 2 survey.

#### Delphi round 1

Potential experts were invited via an email with an embedded “Information Sheet for Participants”, which contained detailed information about the study and a link to “Agree” or “Disagree” to take part in the study. Upon acceptance, experts were asked to consent to participation. Specifically, they were informed that accepting the invitation to participate in the study would constitute implied consent. It was only at this point that the panelists were given access to the online survey to evaluate the set of items presented. This was described as Round 1.

In Round 1, panelists were asked to evaluate items in relation to the safety, efficacy, programming, delivery and mechanism of action of IRT, considering the benefits of IRT compared to other exercise training programs used as nonpharmacological therapy. Items were presented in a randomized order, and panelists graded their opinions of each item using a 5-point Likert scale (strongly disagree (1), disagree (2), do not know (3), agree (4) and strongly agree (5)) to indicate their level of agreement. In addition, panelists were encouraged to provide comments to explain their responses, suggest modifications to any of the items or add further items through free-response text feedback.

Responses from Round 1 were analyzed quantitatively using descriptive statistics for the central tendency (median) and distribution width (interquartile range) to present the collective judgments of the panelists. This information, together with panelists’ deidentified comments, suggested modifications and/or additional items, was provided to the panelists as feedback. The panelists’ comments and suggestions informed Round 2, and the panelists were encouraged to reconsider their opinions on any items that had not reached consensus.

#### Delphi round 2

Panelists who responded to the Round 1 questionnaire were invited to complete Round 2 of the Delphi exercise. For their information, these panelists were sent an email containing a summary of the Round 1 analysis and feedback; they also received the Qualtrics link to the Round 2 questionnaire containing the set of items that did not reach consensus in Round 1. The Round 1 analysis summary comprised the aggregated percentage of agreement for each item along with deidentified suggested modifications and feedback comments from Round 1 and the modified versions of statements where necessary. Each Round 2 statement was either the original or a modified version based on the Round 1 feedback from panelists. Each modified version consisted of the original statement altered by removing, replacing, and/or rephrasing some of the text (indicated by bolding the rephrased or replaced words) or by adding explanatory notes that elaborated further to make the statement clearer and more specific. In this round, items were grouped by their subheadings.

At the end of Round 2, the analysis and feedback processes were repeated with the a priori consensus criteria. Thus, the process yielded two sets of items: one set retained by consensus (experts consistently agreed or consistently disagreed with each item) and another set without a consensus.

### Phase 3: Study team consensus meeting (final item review)

Following Round 2 of the Delphi exercise, all results were discussed by the study team in a final item review. It is common practice to hold a consensus meeting, stakeholder meeting or final item review as the final step of a Delphi study, especially in health care research [62, 72–79]. A similar Delphi approach (two Delphi rounds plus a consensus meeting group), with panel members included in consensus meeting groups, has been reported by other studies [74–77]. The study team members who participated in the final item review are also experts in the area of IRT; all of them have practical experience and publications.

All items on which the expert panelists reached a consensus in both rounds of Delphi exercise were accepted, and items on which the panel did not reach a consensus were discussed in the final item review. Decisions to either accept or reject these remaining items were based on expert panelists’ agreement ratings (≥50% agreement and ≤30% disagreement), consultation of existing guidelines and systematic reviews with meta-analyses, and the team members’ professional experience [64]. The aim was to finalize the content of the list of statements relating to the safety, efficacy, programming, delivery and mechanism of action of IRT.

### Statistical analyses

Descriptive statistics were used to summarize participants’ demographic characteristics (including age, gender, profession, level of education, and years of experience) and panelists’ responses to each statement in the two rounds of the Delphi process. We defined a priori consensus as ≥75% agreement (5-point Likert Scale) with a median ≥4 and interquartile range (IQR) < 2 [80]. For the criterion of ≥75% agreement or disagreement, the 5 levels of the Likert scale were consolidated into 3 categories: “strongly agree/agree”, “strongly disagree/disagree” and “do not know”. Analyses were conducted using IBM SPSS Statistics version 25 for Windows [81].

## Results

This modified Delphi study aimed to investigate expert consensus on the safety, efficacy and delivery of IRT as an adjunct therapy for the management of hypertension.

### Phase 1: Identification of items and expert participants for inclusion

**Fig. 1:**
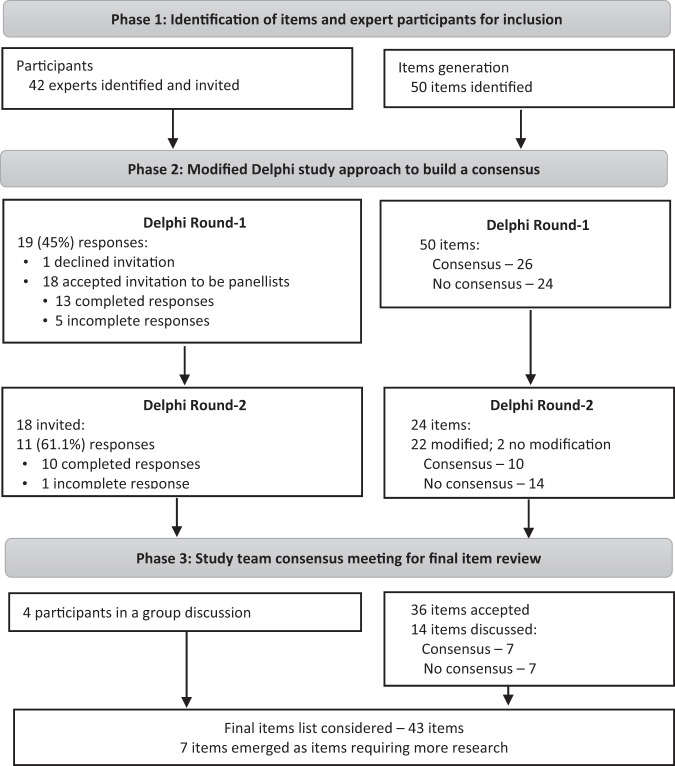
Flow diagram of the items and expert panellists in three phases of the study

A total of 50 items were generated in relation to the safety, efficacy and delivery of IRT. Items were categorized into five subheadings: safety (10 items), efficacy (11 items), programming (12 items), delivery (10 items) and mechanism of action (7 items) (Supplementary Table 1). Forty-two experts were identified based on our predefined inclusion criteria for “expert” panelists; of these eligible experts, 13 and 10 expert panelists fully completed Round 1 and Round 2, respectively. Figure 1 shows a flow diagram of items and panelists through the three phases of the study.

### Phase 2: Modified Delphi study to build a consensus

An invitation to participate in the study was emailed to the 42 potential experts. When the invitation emails were sent, there were no bounced emails or duplicate addresses. However, one address bounced when the reminder email sent to those who did not respond within the three-week period after the first invitation email was sent. There were 19 responses; however, one expert declined to participate. Thus, 18 (43%) experts who met the inclusion criteria consented to participate in the study.

#### Delphi round 1

In Round 1 of the Delphi survey, there were 13 complete and 5 incomplete/partial sets of responses to the questionnaire. The 5 participants with incomplete responses were excluded from the analyses as missing data because they had completed less than 20% of the questionnaire. The remaining 13 panelists, who were included in the analyses, comprised 8 males and 5 females aged between 25 and 64 years with academic/research expertize (9) or clinical expertize (1) and three experts who had both academic and clinical expertize. The demographic profiles of the experts are detailed in Table 1. At the end of Round 1, the panel reached a consensus on 26 (52%) items. These items were not included in Round 2. The 24 items on which the panel did not reach a consensus included items related to safety (8 out of 10 items), efficacy (1 out of 11 items), programming (5 out of 12 items), delivery (6 out of 10 items) and the mechanism of action (4 out of 7 items).

**Table 1:** Demographic characteristics of Delphi participants

Characteristics	Panelists	*n* (%) = 13
Gender	Male	8 (61.5)
	Female	5 (38.5)
Age (years)	25–54	9 (69.2)
	55–64	4 (30.8)
Education	Second degree (Master)/specialization	1 (7.7)
	Third degree (PhD)/sub-specialty	11 (84.6)
	Other (specification) – MD and PhD	1 (7.7)
Profession (by specification)	Clinical exercise physiologist	7 (53.8)
	Academic – Anatomy, Physiology, Pathophysiology	1 (7.7)
	Exercise & sport physician	1 (7.7)
	Exercise scientist	1 (7.7)
	Physical education	1 (7.7)
	Physician- GP specializing in sports and exercise medicine	1 (7.7)
	Professor of Kinesiology	1 (7.7)
Years of professional experience	<5	1 (7.7)
	5–10	6 (46.2)
	11–15	2 (15.4)
	16–20	1 (7.7)
	>20	3 (23.1)
Country of primary work	Australia	2 (15.4)
	Brazil	4 (30.8)
	Canada	2 (15.4)
	Chile	1 (7.7)
	UK	2 (15.4)
	USA	2 (15.4)
Area of primary work	Academic	8 (61.5)
	Hospital/clinic	1 (7.7)
	Sports and exercise facility	1 (7.7)
	Academic and clinical	3 (23.1)
Primary area of expertize for IRT	Academic/research expertize	9 (69.2)
	Clinical expertize	1 (7.7)
	Both	3 (23.1)
For academic/research experts	H-index	
	<10	3 (23.1)
	10–20	3 (23.1)
	21–30	5 (38.5)
	31–39	0
	>40	1 (7.7)
	Publications in total	
	<20	3 (23.1)
	21–50	2 (15.4)
	51–100	3 (23.1)
	>100	4 (30.8)
	Publications in IRT research	
	1	1 (7.7)
	2	3 (23.1)
	3	1 (7.7)
	5 or more	7 (53.8)
For clinical expertize	Years since qualification	
	<5	2 (15.4)
	5–10	1 (7.7)
	>20	1 (7.7)
	Current rank	
	Senior practitioner	1 (7.7)
	Clinical specialist/extended scope practitioner/advanced clinical practice	3 (23.1)

Overall, 31 items were modified (9 items that reached consensus and 22 items to be rerated in Round 2) following the analysis of Round 1 feedback. Ten of the modified versions had explanatory notes added to either the original or a modified statement. The outcome responses for Round 1 are presented by subheading (safety, efficacy, delivery, programming and mechanism of action of IRT) in Supplementary Table 1.

#### Delphi round 2

For Round 2, invitations were sent to 18 panelists, 11 (61%) of whom responded. There was one incomplete response, which was excluded as missing data; thus, 10 panelists’ responses were included in the analysis. In Round 2, the remaining 24 items that did not reach consensus in Round 1 were rerated, with 22 items modified based on suggestions from panelists. At the end of Round 2, an additional 10 (41%) items reached consensus among the panelists, while 14 items failed to reach consensus. The panelists’ responses for Round 2 are summarized in Supplementary Table 1, which includes their agreement in each round for consensus and inclusion in the content list.

### Phase 3: Study team consensus meeting (final item review)

In a final item review, the study team accepted all 36 items that reached consensus in Rounds 1 and 2 of the Delphi study. The remaining 14 items regarding safety (4), efficacy (1), programming (3), delivery (4) and mechanism of action (2) that did not reach consensus at the end of Round 2 were discussed at length. The team accepted 7 of the 14 items and deemed another 7 items inconclusive (i.e., “not accepted”). These were excluded because more research is needed to confirm each item. The team’s decision on these items is documented in Supplementary Table 2.

By the end of the Delphi study, 43 items had reached expert consensus (including two items that negated previous statements) and were accepted for IRT – 7 out of 10 items on safety, 11 out of 11 items on efficacy, 10 out of 12 items on programming, 8 out of 10 items on delivery, and 7 out of 7 items on the mechanism of action. Three items related to safety (2 items for handgrip IRT and one item for all IRT irrespective of leg or handgrip training), 2 items for delivery of leg IRT, and 2 items for programming of handgrip IRT were excluded. Table 2 shows the final list of accepted items, excluding two items (one each for safety and delivery) that negated other statements.

**Table 2:** List of accepted items

No	Item description
Safety	
1	In general, leg IRT employed at an appropriate training intensity (e.g., 20% MVC) causes blood pressure responses of >30 mmHg in SBP or 20 mmHg in DBP.
2	IRT at an appropriate training intensity (e.g., 30% MVC for handgrip or 20% MVC for leg) causes smaller increases in rate pressure product (SBP x HR) compared to moderate intensity aerobic exercise.
3	An appropriate IRT program is safe for people with pre-hypertension.
4	An appropriate IRT program is safe for people with stage 1 hypertension.
5	In general, an appropriate IRT program is safe for people with cardiovascular diseases.
6	An appropriate IRT program is safe for people with peripheral artery disease.
Efficacy	
1	A program of IRT of 8 weeks or longer elicits statistically significant reductions in SBP in healthy individuals.
2	A program of IRT of 8 weeks or longer elicits statistically significant reductions in DBP in healthy individuals.
3	A program of IRT of 8 weeks or longer elicits statistically significant reductions in SBP in cardiovascular disease patients.
4	A program of IRT of 8 weeks or longer elicits statistically significant reductions in DBP in cardiovascular disease risk patients.
5	A program of IRT of 8 weeks or longer elicits clinically meaningful reductions (i.e., ≥2 mmHg reduction) in SBP in healthy individuals.
6	A program of IRT of 8 weeks or longer elicits clinically meaningful reductions (i.e., ≥2 mmHg reduction) in DBP in healthy individuals.
7	A program of IRT of 8 weeks or longer elicits clinically meaningful reductions (i.e., ≥2 mmHg reduction) in SBP in cardiovascular disease patients.
8	A program of IRT of 8 weeks or longer elicits clinically meaningful reductions (i.e., ≥2 mmHg reduction) in DBP in cardiovascular disease patients
9	A program of IRT of 8 weeks or longer elicits SBP and DBP reductions of similar size to those observed with taking one anti-hypertensive medication.
10	A program of IRT of 8 weeks or longer elicits SBP and DBP reductions that are likely to reduce the risk of a serious event related to poor blood pressure control such as stroke and myocardial infarction.
11	An appropriate IRT program may potentially be beneficial for people with heart disease as it elicits an ischaemic pre-conditioning response.
Programming	
1	A typical handgrip IRT program is 4 × 2 min effort at 30% MVC with 1–3 min rest periods in between efforts.
2	A typical leg IRT program is 4 × 2 min effort at 20% MVC with 1–3 min rest periods in between efforts.
3	A typical IRT protocol performed at a minimum frequency of 3 sessions per week for 8 weeks or longer is enough to obtain a clinically meaningful anti-hypertensive response.
4	Prior to IRT programming an individual should be pre-screened including appropriate risk assessment by an appropriate health professional.
5	Ideally, MVC should be measured before every IRT session and training intensity adjusted accordingly to ensure relative effort remains constant.
6	IRT can be used as an alternative form of exercise to lower blood pressure in people with hypertension who are unable to perform other types of exercise (aerobic or dynamic resistance).
7	IRT is relatively simple to perform compared to some other forms of exercise.
8	The handgrip IRT exercise device is portable and simple to transport.
9	Besides walking an IRT program is relatively inexpensive compared to some other forms of exercise.
10	An IRT program takes less time to perform and to elicit anti-hypertensive benefits than other types of exercise.
Delivery	
1	Handgrip IRT is simple to deliver as one can measure MVC and then prescribe IRT at 30% MVC and this may also reduce barriers to dynamic exercise training.
2	Handgrip IRT below 5% MVC is unlikely to elicit anti-hypertensive benefits.
3	Leg IRT is more difficult to prescribe as opposed to handgrip IRT as sophisticated laboratory/gym equipment is required to determine MVC for leg IRT.
4	Handgrip IRT performed at MVC above 30% is sub-optimal as it increases the risk of exaggerated blood pressure responses and reduces the ability of people to complete their handgrip program.
5	Handgrip IRT employed at an appropriate training intensity can be prescribed by a qualified exercise specialist for home-based training with little or no supervision – using the appropriate risk assessment prior to training.
6	Leg IRT employed at an exact training intensity of 20% MVC can be prescribed by a qualified exercise specialist for home-based training with little or no supervision.
7	Prescription of handgrip IRT is preferred to leg IRT for home-based delivery as the former is inexpensive and easier to use compared to the later which requires sophisticated laboratory/gym equipment to determine precise MVC.
Mechanism of action	
1	A possible mechanism for IRT to work is via repeated exposure to blood vessel occlusion that causes shear stress on the arterial wall with a resultant increase in nitric oxide release triggering vasodilation.
2	Repeated exposure of IRT may cause permanent changes in blood vessels (e.g., diameter) with time. Longer term (i.e., ≥6 months) might lead to more consistent blood pressure adaptations.
3	IRT generates reactive hyperaemia which is facilitated by vasodilation.
4	Repeated exposure to IRT increases baroreflex sensitivity which may improve cardiac autonomic modulation.
5	Depending on the length of the protocol, the anti-hypertensive effects of IRT are reversed within 2–5 weeks detraining.
6	The anti-hypertensive effects of IRT are transient if the individual discontinues the training.
7	The anti-hypertensive effects of IRT are semi-permanent (e.g., lasting up to 4 weeks) if training is discontinued.

Note: Two items that negated a previous statement have been excluded from accepted items

## Discussion

This study examines pertinent issues regarding the safety, efficacy, and delivery of IRT. This is the first formal consensus-building exercise based upon items generated from evidence-based literature on the antihypertensive effects of IRT, and this study involved experts with both academic and clinical experience in this field of research. IRT is an emerging antihypertensive therapy; given this status, concerns may remain around matters such as safety, efficacy and delivery. We therefore compiled a set of 50 items considered important in relation to the safety, efficacy, programming, delivery and mechanism of action of IRT. This modified Delphi study called on the expertize and reflection of the panelists and had them rate their agreement on the use and antihypertensive effect of IRT.

Round 1 feedback from panelists informed modifications to some items for rerating in Round 2 as well as some items that had already reached consensus in Round 1. These modifications were minor and made the statements clearer and more specific. In two items, the original statements were negated by adding “does not”. The main purpose of this reversal was to substantiate the validity of panelists’ responses with reference to the original statements (we expected the opposites of their original responses). After Round 1 and Round 2, a total of 36 items (out of 50) achieved ≥75% consensus among the expert panelists. The remaining 14 items, with <75% consensus, were discussed by the study team for either inclusion (7 items) or exclusion (7 items) in the final item review, considering the comments from the panelists as well as evidence-based research and guidelines.

The present Delphi study identified a set of 43 out of 50 statements that support the use of IRT as an adjunct therapy for the control of blood pressure in both healthy people and those living with hypertension. All items related to the efficacy and mechanism of action of IRT reached expert consensus; only a few items related to safety (3), delivery (2) and programming (2) failed to reach consensus. Despite the previous concerns addressed by Hansen et al. [56] and Pescatello et al. [58] regarding the use of IRT in clinical practice, this study showed consensus (in consultation with evidence-based literature) on the benefits of IRT as an adjunct treatment for hypertension. This Delphi study is in agreement with the findings of Wiles et al. [82], Carlson et al. [83] and Smart et al. [9, 44] regarding the safety of IRT and the findings of Herrod et al. [35], Carlson et al. [84] and Wiles et al. [85] on its efficacy. The results are also supported by Lopez-Valenciano et al. [43] and Punia et al. [86] concerning the potential use of IRT as an adjunct to lowering BP. Likewise, larger and more robust meta-analyses [42, 44] and current empirical studies [45, 46, 53, 59–61] demonstrate the safety and effectiveness of IRT in preventing hypertension. In addition, the findings show that the set of items is comprehensive, as the panelists suggested no additional items. Even though some comments and feedback were received from panelists, this was to justify their response and to indicate areas that needed more research. The remaining 7 items were excluded from the content, representing emerging areas for future research. These 7 items included 3 items related to safety, 2 items related to delivery, and 2 items related to programming (see Supplementary Table 2). Regarding the safety of IRT, 3 items relating to increases in BP and HR did not reach consensus. It is important to note that blood pressure usually increases with exercise as a result of the increased oxygen demand of working muscles; mechanistically, this rise in blood pressure occurs via increased sympathetic tone, which results in increased cardiac output [87]. During exercise, it is normal for some individuals to have an exaggerated blood pressure increase, which usually returns to baseline levels after exercise irrespective of the type of exercise [88]. This phenomenon is known as a hypertensive response to exercise (HRE), and there is no consensus on the exact extent of HRE in IRT [87–90]. O’Driscoll et al. [91] recently showed that BP increased significantly from baseline at the onset of isometric exercise and remained elevated during the exercise session; afterward, recovery resulted in a significant reduction in BP, even below baseline. Although HRE is considered an irregular response, the variable increase differs among individuals in a manner that suggests the partial influence of genetic variation [92–95]. This characterizes HRE as a physiological phenomenon affected by the interaction of both central and reflex neural control mechanisms of the cardiovascular system during exercise [88] as well as the individual’s baseline characteristics [96]. Nonetheless, sympathetic nervous system stimulation and the renin-angiotensin-aldosterone system have been associated with increases in blood pressure and heart rate during exercise [87, 88, 90]. Likewise, impaired endothelial vasodilation may contribute to HRE [97, 98]. The majority of work regarding the effect of IRT on blood pressure has focused on the acute and chronic postexercise effects and not on the blood pressure response during exercise. Wiles et al. [82] ascertained the safety of IRT in stage 1 hypertensive patients by investigating the hemodynamic response during isometric wall squat exercise and isometric wall squat training sessions. These authors found that blood pressure and heart rate during isometric exercise were considerably lower than the thresholds recommended by the American College of Sports Medicine (ACSM) guidelines for exercise test termination [99]. Moreover, their highest recorded rate-pressure product (20,681 ± 3911 mmHg × bpm) was much less than that reported (27,729 ± 5018 mmHg × bpm) in high-risk patients referred for exercise stress testing (using the Bruce protocol on a treadmill) for ischemic heart disease evaluation [100]. To date, no study has specifically investigated blood pressure responses during IRT compared with other types of exercise (especially aerobic exercise training or dynamic resistance training) in both healthy individuals and those with chronic diseases. However, Seidel et al. [101] have recently reported the differential impacts of AET and IRT on blood pressure variability and central aortic blood pressure. The parameters measured in that study addressed the cumulative effects of exercise after the end of training but not during training. Therefore, further studies are needed to quantify the hemodynamic responses that occur during IRT.

Regarding the delivery and programming of IRT, the four items excluded from the content were all related to leg IRT. The reason why consensus was elusive is likely because more studies involved handgrip IRT than leg IRT. Typically, for targeted leg IRT, a leg extension dynamometer is required; this is usually gymnasium/laboratory equipment used to determine the intensity of maximal voluntary contraction. However, there are other forms of leg IRT that have had similar beneficial effects when performed by normotensive [85, 91], prehypertensive [102] and stage 1 hypertensive patients [82, 103] in the context of wall squat exercises. These require a specific degree of knee joint flexion, which can be determined by using a simple tool—a modified clinical goniometer secured at the knee using four 25-mm-wide elastic Velcro leg straps [82]. Similarly, in home-based exercise training, an alternative device was used; this device, the “Bend and Squat” (an improvised tool made in house), aligns a participant’s feet and back into the correct position for a given knee joint angle during wall squats [85]. There is still more work to be done in order to quantify the individual’s perceived difficulty ratings and adherence to leg IRT for home-based training compared to handgrip IRT and/or other modes of exercise to confirm any apprehensions. Recent work by Lea et al. [104] showed that the rating of perceived exertion assessed across consecutive workloads and time points provides a valid and reliable measure of IRT intensity and physiological exertion.

### Study highlights and the way forward

The adoption of IRT as an adjunct therapy remains underutilized in clinical practice and is omitted from some guidelines on hypertension management. The consensus achieved in the current study highlights the safety, efficacy and delivery of IRT, as these are vital for translation into clinical practice. In addition, statements regarding the programming and mechanism of action of IRT reached consensus. There is substantial evidence to support the benefits of IRT in blood pressure control; however, large-scale clinical trials are warranted to foster adoption.

Future research should focus on the following:

1. Real-time hemodynamic studies of blood pressure during IRT compared with other types of exercise (especially aerobic or dynamic resistance training) in healthy participants and people with chronic diseases to quantify the effects during training;
2. Rating individuals’ perceptions of the difficulty of leg IRT and adherence to their regimens for home-based training compared to handgrip IRT and/or other modes of exercise to quantify any apprehension in relation to delivery and adherence; and
3. Comparing the effect of leg and handgrip IRT to quantify blood pressure and hemodynamic responses.

### Strengths and limitations

This study has a number of strengths. The literature search for item generation included systematic reviews, from which individual studies were further scrutinized for the effect of IRT on blood pressure response parameters. A modified Delphi technique for consensus building preserved the anonymity of panelists, allowing them to express their opinions freely without any restrictions. This reduced the influence of dominant personalities and/or panel experts’ status on the results. It also allowed panel members to make suggestions and provide comments on the potential modification or addition of items. More importantly, this technique is inexpensive and flexible, allowing experts to contribute irrespective of their geographical location without needing to make physical contact. There was diversity in experts’ professional backgrounds in relation to exercise and sport science, spanning from academic to clinical practice, with all panelists having at least a graduate degree and years of experience. The majority of the panelists with academic/research expertize had 5 or more publications in IRT research, while those with clinical expertize were ranked as senior practitioners or were in advanced clinical practice and had more than 5 years of experience. However, this study is not free of limitations. The overall response rate in Round 1 (19 out of 42 invited experts—45%) was low, with 13 out of 19 participants submitting complete responses. This further reduced in Round 2, with 11 out of 18 invited experts responding and 10 submitting complete responses, thus limiting the number of experts involved in making the decisions. The reasons for low respondent rates cannot be explained but could be related to COVID-19 and may include time management issues. The Qualtrics survey could have been set up with a longer active response period to allow more time for completion. This could potentially have compensated for data lost due to incomplete responses.

## Conclusion

This study undertook a modified Delphi process to reach an expert consensus on the safety, efficacy and delivery of IRT as an adjunct therapy in the management of hypertension. Experts reached consensus on the efficacy and mechanism of action of IRT. Some concerns remain in the area of IRT safety and, to a minor extent, in optimal programming and delivery of leg IRT. Future research in these areas is needed to allay concerns and to fully translate IRT into clinical practice.

## Supplementary information

SUPPLEMENTARY FILES
