# Supplementary material for: Safety, efficacy and delivery of isometric resistance training as an adjunct therapy for blood pressure control: a modified Delphi study
Source: Hypertens Res. 2022 Jan 12;45(3):483–95. doi: 10.1038/s41440-021-00839-3 (PMC8752388; doi:10.1038/s41440-021-00839-3)
Supplement: Supplementary file 1 — SUPPLEMENTARY FILES [file 41440_2021_839_MOESM1_ESM.docx]

Safety, efficacy and delivery of isometric resistance training as an adjunct therapy for blood pressure control: A modified Delphi study.

Biggie Baffour-Awuah, Melissa J Pearson, Neil A Smart, Gudrun Dieberg

**SUPPLEMENTARY FILES**

**Supplementary** **Table 1** Summary of Delphi Round-1 and 2 agreement rating and decision

| **Item code**  **category** | **Item description** | **ROUND-1** | | | | | | | **New Item code** | **Modification of items** | **ROUND-2** | | | | | | |
| --- | --- | --- | --- | --- | --- | --- | --- | --- | --- | --- | --- | --- | --- | --- | --- | --- | --- |
|  |  | **Strongly agree and agree (%)** | **Strongly disagree and disagree (%)** | **Don’t know (%)** | **Median rating** | **Inter quartile range** | **25th percentile** | **75th percentile** |  |  | **Strongly agree and agree (%)** | **Strongly disagree and disagree (%)** | **Don’t know (%)** | **Median** | **Inter quartile range** | **25th percentile** | **75th percentile** |
| **IRTQ1**  Safety  S | Handgrip IRT employed at an appropriate training intensity (e.g. 30% MVC) causes exaggerated blood pressure responses (either increases >30mmHg in SBP or 20mmHg in DBP). | 30.8 | 61.5 | 7.7 | 1  SD | 3 | 1  SD | 4  A | **IRT-2Q1** | Handgrip IRT employed at an appropriate training intensity (e.g. 30% MVC) causes blood pressure responses of >30mmHg in SBP or 20mmHg in DBP.  ***Note:*** *- Depending on the exercise, intensity BP increases of 30 – 50 mmHg are quite common in aerobic or dynamic resistance training.* | 40 | 60 | 0 | 2  D | 3.25 | 1  SD | 4.25  A-SA |
|  |  |  |  |  |  |  |  |  |  |  | Non-consensus  Study team to make final decision  (see Table S2) | | | | | | |
| **IRTQ2**  Efficacy | A program of IRT of 8 weeks or longer elicits statistically significant reductions in SBP in cardiovascular disease risk patients. | 92.3 | 0.0 | 7.7 | 5  SA | 1 | 4  A | 5  SA |  | A program of IRT of 8 weeks or longer elicits statistically significant reductions in SBP in **cardiovascular disease patients.** | Included in the final content list after round-1 | | | | | | |
| **IRTQ3**  Delivery | Handgrip IRT is simple to deliver as one can measure MVC and then prescribe IRT at 30% MVC. | 100 | 0.0 | 0.0 | 5  SA | 1 | 4  A | 5  SA |  | Handgrip IRT is simple to deliver as one can measure MVC and then prescribe IRT at 30% MVC **and this may also reduce barriers to dynamic exercise training.** | Included in the final content list after round-1 | | | | | | |
| **IRTQ4**  Programming | A typical handgrip IRT program is 4x2 min effort at 30% MVC with 1-3 min rest periods in between efforts. | 100 | 0% | 0.0 | 5  SA | 0 | 5  SA | 5  SA |  | No modification | Included in the final content list after round-1 | | | | | | |
| **IRTQ5**  Mechanism | IRT works via repeated exposure to blood vessel occlusion that causes shear stress on the arterial wall with a resultant increase in nitric oxide release triggering vasodilation. | 69.2% | 15.4% | 15.4% | 4  A | 1 | 3  DK | 4  A | **IRT-2Q21** | **A possible mechanism for IRT to work is** via repeated exposure to blood vessel occlusion that causes shear stress on the arterial wall with a resultant increase in nitric oxide release triggering vasodilation. | 100 | 0 | 0 | 4  A | 1 | 4  A | 5  SA |
|  |  |  |  |  |  |  |  |  |  |  | Included | | | | | | |
| **IRTQ6**  Safety | An appropriate IRT program is safe for people with pre-hypertension. | 15.4 | 0.0 | 84.6 | 3  DK | 0 | 3  DK | 3  DK | **IRT-2Q2** | An appropriate IRT program is safe for people with pre-hypertension.  ***Note: -*** *You all agreed IRT program is safe for people with stage 1 hypertension.* | 100 | 0.0 | 0.0 | 5  SA | 0 | 5  SA | 5  SA |
|  |  |  |  |  |  |  |  |  |  |  | Included | | | | | | |
| **IRTQ7**  Efficacy | An appropriate IRT program is beneficial for people with heart disease as it elicits an ischaemic pre-conditioning response. | 53.8 | 15.4 | 30.8 | 4  A | 2 | 3  DK | 5  SA | **IRT-2Q9** | An appropriate IRT program **may potentially be** beneficial for people with heart disease as it elicits an ischaemic pre-conditioning response. | 70 | 10 | 20 | 4  A | 1 | 3  DK | 4  A |
|  |  |  |  |  |  |  |  |  |  |  | Non-consensus  Study team to make final decision  (see Table S2) | | | | | | |
| **IRTQ8**  Delivery | Handgrip IRT below 10% MVC is unlikely to elicit anti-hypertensive benefits. | 53.8 | 23.1 | 23.1 | 4  A | 2.5 | 2.5  D-DK | 5  SA | **IRT-2Q10** | Handgrip IRT below **5%** MVC is unlikely to elicit anti-hypertensive benefits. | 100 | 0 | 0 | 4.5  A-SA | 1 | 4  A | 5  SA |
|  |  |  |  |  |  |  |  |  |  |  | Included | | | | | | |
| **IRTQ9**  Programming | A typical IRT protocol performed at a minimum frequency of 3 sessions per week for 8 weeks or longer is enough to obtain a clinically meaningful anti-hypertensive response. | 46.2 | 0.0 | 53.8 | 3  DK | 2 | 3  DK | 5  SA | **IRT-2Q16** | A typical IRT protocol performed at a minimum frequency of 3 sessions per week for 8 weeks or longer is enough to obtain a clinically meaningful anti-hypertensive response.  ***Note:*** *- You all agreed earlier for efficacy.* | 100 | 0 | 0 | 4.5  A-SA | 1 | 4  A | 5  SA |
|  |  |  |  |  |  |  |  |  |  |  | Included | | | | | | |
| **IRTQ10**  Mechanism | Repeated exposure of IRT can cause permanent changes in blood vessel diameter with time. | 38.5 | 15.4 | 46.2 | 3  DK | 1 | 3  DK | 4  A | **IRT-2Q22** | Repeated exposure of IRT **may** cause permanent changes in blood vessels **(e.g. diameter)** with time. **Longer term (i.e. ≥6 months) might lead to more consistent blood pressure adaptations.** | 70 | 10 | 20 | 4  A | 1 | 3  DK | 4  A |
|  |  |  |  |  |  |  |  |  |  |  | Included | | | | | | |
| **IRTQ11**  Safety | Leg IRT employed at an appropriate training intensity (e.g. 20% MVC) causes blood pressure responses (either increases >30mmHg in SBP or 20mmHg in DBP). | 38.5 | 30.8 | 30.8 | 3  DK | 2 | 2  D | 4  A | **IRT-2Q3** | **In general,** leg IRT employed at an appropriate training intensity (e.g. 20% MVC) causes **blood pressure responses of >30mmHg in SBP or 20mmHg in DBP.**  ***Note:*** *- Depending on the exercise intensity BP increases of 30 – 50 mmHg are quite common in aerobic and dynamic resistance training.* | 80 | 20 | 0 | 4  A | 1.5 | 3.5  DK-A | 5  SA |
|  |  |  |  |  |  |  |  |  |  |  | Included | | | | | | |
| **IRTQ12**  Efficacy | A program of IRT of 8 weeks or longer elicits statistically significant reductions in DBP in cardiovascular disease risk patients. | 76.9 | 7.7 | 15.4 | 4  A | 1.5 | 3.5  DK-A | 5  SA |  | No modification | Included in the final content list after round-1 | | | | | | |
| **IRTQ13**  Delivery | Handgrip IRT is difficult to prescribe as sophisticated laboratory/gym equipment is required to determine MVC. | 23.1 | 76.9 | 0.0 | 1  SD | 2 | 1  SD | 3  DK |  | No modification | Included in the final content list after round-1 | | | | | | |
| **IRTQ14**  Programming | A typical leg IRT program is 4x2 min effort at 20% MVC with 1-3 min rest periods in between efforts. | 76.9 | 7.7 | 15.4 | 4  A | 1.5 | 3.5  DK-A | 5  SA |  | No modification | Included in the final content list after round-1 | | | | | | |
| **IRTQ15**  Mechanism | IRT generates reactive hyperaemia which is facilitated by vasodilation. | 92.3 | 0.0 | 7.7 | 4  A | 0.5 | 4  A | 4.5  A-SA |  | No modification | Included in the final content list after round-1 | | | | | | |
| **IRTQ16**  Safety | IRT employed at an appropriate training intensity (e.g. 30% MVC for handgrip or 20% MVC for leg) causes increases in heart rate >15 beats per minute. | 69.2 | 15.4 | 15.4 | 4  A | 1 | 3  DK | 4  A | **IRT-2Q4** | IRT employed at an appropriate training intensity (e.g. 30% MVC for handgrip or 20% MVC for leg) causes increases in heart rate **response of** >15 beats per minute.  ***Note: -*** *Depending on the exercise intensity HR response increases >20 bpm as seen in aerobic and dynamic resistance training.* | 60 | 40 | 0 | 4  A | 3 | 1  SD | 4  A |
|  |  |  |  |  |  |  |  |  |  |  | Non-consensus  Study team to make final decision  (see Table S2) | | | | | | |
| **IRTQ17**  Efficacy | A program of IRT of 8 weeks or longer elicits clinically meaningful reductions (i.e. ≥2mmHg reduction) in SBP in cardiovascular disease risk patients. | 100 | 0.0 | 0.0 | 5  SA | 1 | 4  A | 5  SA |  | A program of IRT of 8 weeks or longer elicits clinically meaningful reductions (i.e. ≥2mmHg reduction) in SBP in cardiovascular disease patients. | Included in the final content list after round-1 | | | | | | |
| **IRTQ18**  Delivery | Leg IRT is simple to deliver as no equipment is required. | 38.5 | 46.2 | 15.4 | 3  DK | 2 | 2  D | 4  A | **IRT-2Q11** | Leg IRT **at 20%** **MVC** is simple to deliver as no equipment is required. | 50 | 30 | 20 | 3.5  DK-A | 2.25 | 2  D | 4.25  A-SA |
|  |  |  |  |  |  |  |  |  |  |  | Non-consensus  Study team to make final decision  (see Table S2) | | | | | | |
| **IRTQ19**  Programming | Prior to IRT programming an individual should be pre-screened by an appropriate health professional. | 84.6 | 7.7 | 7.7 | 4  A | 1 | 4  A | 5  SA |  | Prior to IRT programming an individual should be pre-screened **including appropriate risk assessment** by an appropriate health professional. | Included in the final content list after round-1 | | | | | | |
| **IRTQ20**  Mechanism | Repeated exposure to IRT increases baroreflex sensitivity which improves cardiac autonomic modulation. | 61.5 | 7.7 | 30.8 | 4  A | 1 | 3  DK | 4  A | **IRT-2Q23** | Repeated exposure to IRT increases baroreflex sensitivity which **may** improve cardiac autonomic modulation. | 70 | 0 | 30 | 4  A | 1 | 3  DK | 4  A |
|  |  |  |  |  |  |  |  |  |  |  | Non-consensus  Study team to make final decision  (see Table S2) | | | | | | |
| **IRTQ21**  Safety | IRT at an appropriate training intensity (e.g. 30% MVC for handgrip or 20% MVC for leg) causes smaller increases in rate pressure product (SBP x HR) compared to moderate intensity aerobic exercise. | 53.8 | 15.4 | 30.8 | 4  A | 2 | 3  DK | 5  SA | **IRT-2Q5** | IRT at an appropriate training intensity (e.g. 30% MVC for handgrip or 20% MVC for leg) causes smaller increases in rate pressure product (SBP x HR) compared to moderate intensity aerobic exercise.  ***Note:*** *- the highest RPP after IRT reported by Carlson et al. 2017 in pre-hypertensive (14136±5879 mmHgbpm)^31^ and Wiles et al. 2018 in stage 1 hypertensive population (20681±3911 mm Hgbpm)^47^ compared to that reported (27,729±5018 mmHgbpm) in high risk patients referred for clinical exercise testing for the evaluation of ischaemic heart disease^48^.* | 80 | 20 | 0 | 4.5  A-SA | 1.75 | 3.25  DK-A | 5  SA |
|  |  |  |  |  |  |  |  |  |  |  | Included | | | | | | |
| **IRTQ22**  Efficacy | A program of IRT of 8 weeks or longer elicits SBP and DBP reductions that are likely to reduce the risk of a serious event related to poor blood pressure control such as stroke and myocardial infarction. | 84.6 | 7.7 | 7.7 | 5  SA | 1 | 4  A | 5  SA |  | No modification | Included in the final content list after round-1 | | | | | | |
| **IRTQ23**  Delivery | Leg IRT can be accurately delivered at a precise MVC intensity. | 38.5 | 38.5 | 23.1 | 3  DK | 2 | 2  D | 4  A | **IRT-2Q12** | Leg IRT can be accurately delivered at a precise MVC intensity **without the use of gym/laboratory equipment.** | 30 | 30 | 40 | 3  DK | 2 | 2  D | 4  A |
|  |  |  |  |  |  |  |  |  |  |  | Non-consensus  Study team to make final decision  (see Table S2) | | | | | | |
| **IRTQ24**  Programming | MVC should ideally be measured before every IRT session and training intensity adjusted accordingly so relative effort remains constant. | 69.2 | 30.8 | 0.0 | 4  A | 3 | 2  D | 5  SA | **IRT-2Q17** | **Ideally**, MVC should be measured before every IRT session and training intensity adjusted accordingly **to ensure** relative effort remains constant. | 80 | 10 | 10 | 4.5  A-SA | 1.25 | 3.75  DK-A | 5  SA |
|  |  |  |  |  |  |  |  |  |  |  | Included | | | | | | |
| **IRTQ25**  Mechanism | The anti-hypertensive effects of IRT are reversed within 2-4 weeks detraining. | 46.2 | 23.1 | 30.8 | 3  DK | 1.5 | 2.5  D-DK | 4  A | **IRT-2Q24** | **Depending on the length of the protocol,** the anti-hypertensive effects of IRT are reversed within 2 – 5 weeks detraining.  ***Note:*** *Detraining times for aerobic exercise (2 – 4 weeks*) | 80 | 0 | 20 | 4  A | 0.5 | 3.75  DK-A | 4.25  A-SA |
|  |  |  |  |  |  |  |  |  |  |  | Included | | | | | | |
| **IRTQ26**  Safety | An appropriate IRT program is safe for people with stage 1 hypertension. | 100 | 0.0 | 0.0 | 5  SA | 1 | 4  A | 5  SA |  | No modification | Included in the final content list after round-1 | | | | | | |
| **IRTQ27**  Efficacy | A program of IRT of 8 weeks or longer elicits statistically significant reductions in SBP in healthy individuals. | 84.6 | 15.4 | 0.0 | 5  SA | 1 | 4  A | 5  SA |  | No modification | Included in the final content list after round-1 | | | | | | |
| **IRTQ28**  Delivery | Handgrip IRT performed at MVC above 30% is sub-optimal as it increases the risk of exaggerated blood pressure responses and reduces the ability of people to complete their handgrip program. | 61.5 | 38.5 | 0.0 | 4  A | 3 | 2  D | 5  SA | **IRT-2Q13** | Handgrip IRT performed at MVC above 30% is sub-optimal as it increases the risk of exaggerated blood pressure responses and reduces the ability of people to complete their handgrip program.  ***Note:*** *- Intensities at >30% MVC may cause participants to experience significant discomfort which could discourage adherence.* | 80 | 10 | 10 | 4  A | 0.5 | 3.75  DK-A | 4.25  A-SA |
|  |  |  |  |  |  |  |  |  |  |  | Included | | | | | | |
| **IRTQ29**  Programming | Due to its simplicity a handgrip IRT program prescribed for home-based training will most likely result in higher adherence than an aerobic and/or strength exercise program. | 69.2 | 23.1 | 7.7 | 4  A | 2 | 2.5  D-DK | 4.5  A-SA | **IRT-2Q18** | No modification | 60 | 20 | 20 | 4  A | 2.25 | 2.75  D-DK | 5  SA |
|  |  |  |  |  |  |  |  |  |  |  | Non-consensus  Study team to make final decision  (see Table S2) | | | | | | |
| **IRTQ30**  Mechanism | The anti-hypertensive effects of IRT are transient if the individual discontinues the training. | 92.3 | 7.7 | 0.0 | 4  A | 1 | 4  A | 5  SA |  | No modification | Included in the final content list after round-1 | | | | | | |
| **IRTQ31**  Safety | An appropriate IRT program is safe for people with heart failure. | 61.5 | 0.0 | 38.5 | 4  A | 1 | 3  DK | 4  A | **IRT-2Q6** | **In general,** an appropriate IRT program is safe for people with **cardiovascular diseases**. | 100 | 0 | 0 | 4.5  A-SA | 1 | 4  A | 5  SA |
|  |  |  |  |  |  |  |  |  |  |  | Included | | | | | | |
| **IRTQ32**  Efficacy | A program of IRT of 8 weeks or longer elicits clinically meaningful reductions (i.e. ≥2mmHg reduction) in DBP in cardiovascular disease risk patients. | 84.6 | 0.0 | 15.4 | 4  A | 1 | 4  A | 5  SA |  | A program of IRT of 8 weeks or longer elicits clinically meaningful reductions (i.e. ≥2mmHg reduction) in DBP in **cardiovascular disease patients.** | Included in the final content list after round-1 | | | | | | |
| **IRTQ33**  Delivery | Leg IRT is more difficult to prescribe as opposed to handgrip IRT as sophisticated laboratory/gym equipment is required to determine MVC for leg IRT. | 69.2 | 15.4 | 15.4 | 4  A | 1.5 | 3  DK | 4.5  A-SA | **IRT-2Q14** | No modification | 50 | 30 | 20 | 3.5  DK-A | 2.25 | 1.75  SD-D | 4  A |
|  |  |  |  |  |  |  |  |  |  |  | Non-consensus  Study team to make final decision  (see Table S2) | | | | | | |
| **IRTQ34**  Programming | IRT can be used as an alternative form of exercise to lower blood pressure in people with hypertension who are unable to perform other types of exercise. | 92.3 | 7.7 | 0.0 | 5  A | 1 | 4  A | 5  SA |  | IRT can be used as an alternative form of exercise to lower blood pressure in people with hypertension who are unable to perform other types of exercise **(aerobic or dynamic resistance)**. | Included in the final content list after round-1 | | | | | | |
| **IRTQ35**  Mechanism | The anti-hypertensive effects of IRT are semi-permanent (e.g. lasting up to 4 weeks) if training is discontinued. | 76.9 | 0.0 | 23.1 | 4  A | 0.5 | 3.5  DK-A | 4  SA |  | No modification | Included in the final content list after round-1 | | | | | | |
| **IRTQ36**  Safety | Handgrip IRT employed at an appropriate training intensity (e.g. 30% MVC) does not cause exaggerated blood pressure responses (either increases >30mmHg in SBP or 20mmHg in DBP). | 61.5 | 23.1 | 15.4 | 4  A | 2 | 2.5  D-DK | 4.5  A-SA | **IRT-2Q7** | Handgrip IRT employed at an appropriate training intensity (e.g. 30% MVC) does not cause blood pressure responses of >30mmHg in SBP or 20mmHg in DBP.  ***Note:*** *- Depending on the intensity BP increases of 30 – 50 mmHg are quite common in aerobic or dynamic resistance training.* | 60 | 30 | 10 | 4  A | 3 | 2  D | 5  SA |
|  |  |  |  |  |  |  |  |  |  |  | Non-consensus  Study team to make final decision  (see Table S2) | | | | | | |
| **IRTQ37**  Efficacy | A program of IRT of 8 weeks or longer elicits clinically meaningful reductions (i.e. ≥2mmHg reduction) in DBP in healthy individuals. | 76.9 | 23.1 | 0.0 | 4  A | 2 | 3  DK | 5  SA |  | No modification | Included in the final content list after round-1 | | | | | | |
| **IRTQ38**  Delivery | Handgrip IRT employed at an appropriate training intensity can be prescribed by a qualified exercise specialist for home-based training with little or no supervision. | 92.3 | 0.0 | 7.7 | 4  A | 1 | 4  A | 5  SA |  | Handgrip IRT employed at an appropriate training intensity can be prescribed by a qualified exercise specialist for home-based training with little or no supervision – **using the appropriate risk assessment prior to training.** | Included in the final content list after round-1 | | | | | | |
| **IRTQ39**  Programming | Due to its simplicity a leg IRT program prescribed for home-based training will most likely result in a higher adherence than an aerobic and/or strength exercise program. | 23.1 | 15.4 | 61.5 | 3  DK | 0.5 | 3  DK | 3.5  DK-A | **IRT-2Q19** | **Compared to handgrip IRT, a leg IRT program at 20% MVC** prescribed for home-based training will most likely result in a higher adherence than an aerobic and/or strength exercise program **due to its** **simplicity**. | 20 | 10% | 70% | 3  DK | 0.25 | 3  DK | 3.25  DK-A |
|  |  |  |  |  |  |  |  |  |  |  | Non-consensus  Study team to make final decision  (see Table S2) | | | | | | |
| **IRTQ40**  Safety | An appropriate IRT program is safe for people with peripheral artery disease. | 84.6 | 0.0 | 15.4 | 4  A | 0.5 | 4  A | 4.5  A-SA |  | No modification | Included in the final content list after round-1 | | | | | | |
| **IRTQ41** Efficacy | A program of IRT of 8 weeks or longer elicits statistically significant reductions in DBP in healthy individuals. | 76.9 | 23.1 | 0.0 | 4  A | 2 | 3  DK | 5  SA |  | No modification | Included in the final content list after round-1 | | | | | | |
| **IRTQ42**  Delivery | Leg IRT employed at appropriate training intensity can be prescribed by a qualified exercise specialist for home-based training with little or no supervision. | 76.9 | 7.7 | 15.4 | 4  A | 1 | 3.5  DK-A | 4.5  A-SA |  | Leg IRT employed **at an exact** training intensity **of 20% MVC** can be prescribed by a qualified exercise specialist for home-based training with little or no supervision. | Included in the final content list after round-1 | | | | | | |
| **IRTQ43**  Programming | IRT is relatively simple to perform compared to some other forms of exercise. | 84.6 | 15.4 | 0.0 | 4  A | 0.5 | 4  A | 4.5  A-SA |  | No modification | Included in the final content list after round-1 | | | | | | |
| **IRTQ44**  Safety | Leg IRT employed at an appropriate training intensity (e.g. 20% MVC) does not cause exaggerated blood pressure responses (either increases >30mmHg in SBP or 20mmHg in DBP). | 23.1 | 46.2 | 30.8 | 3  DK | 1.5 | 2  D | 3.5  DK-A | **IRT-2Q8** | Leg IRT employed at an appropriate training intensity (e.g. 20% MVC) does not cause blood pressure responses of >30mmHg in SBP or 20mmHg in DBP.  ***Note:*** *- Depending on the intensity BP increases of 30 – 50 mmHg are quite common in aerobic or dynamic resistance training.* | 20 | 70 | 10 | 2  D | 2.25 | 1  SD | 3.25  DK-A |
|  |  |  |  |  |  |  |  |  |  |  | Non-consensus  Study team to make final decision  (see Table S2) | | | | | | |
| **IRTQ45**  Efficacy | A program of IRT of 8 weeks or longer elicits SBP and DBP reductions of similar size to those observed with taking one anti-hypertensive medication. | 84.6 | 15.4 | 0.0 | 4  A | 0 | 4  A | 4  A |  | No modification | Included in the final content list after round-1 | | | | | | |
| **IRTQ46**  Delivery | Prescription of handgrip IRT is preferred to leg IRT for home-based delivery as the former does not require sophisticated laboratory/gym equipment to determine MVC. | 53.8% | 23.1% | 23.1% | 4  A | 1.5 | 2.5  D-DK | 4  A | **IRT-2Q15** | Prescription of handgrip IRT is preferred to leg IRT for home-based delivery as the former **is inexpensive and easier to use compared to the later which** requires sophisticated laboratory/gym equipment to determine **precise** MVC. | 50 | 30 | 20 | 3.5  DK-A | 2 | 2  D | 4  A |
|  |  |  |  |  |  |  |  |  |  |  | Non-consensus  Study team to make final decision  (see Table S2) | | | | | | |
| **IRTQ47**  Programming | The handgrip IRT exercise device is portable and simple to transport. | 100% | 0% | 0% | 5  SA | 0.5 | 4.5  A-SA | 5  SA |  | No modification | Included in the final content list after round-1 | | | | | | |
| **IRTQ48** Efficacy | A program of IRT of 8 weeks or longer elicits clinically meaningful reductions (i.e. ≥2mmHg reduction) in SBP in healthy individuals. | 84.6 | 15.4 | 0.0 | 5  SA | 1 | 4  A | 5  SA |  | No modification | Included in the final content list after round-1 | | | | | | |

| **IRTQ49**  Programming | An IRT program is relatively inexpensive compared to some other forms of exercise. | 61.5 | 30.8 | 7.7 | 4  A | 3 | 2  D | 5  SA | **IRT-2Q20** | **Besides walking** an IRT program is relatively inexpensive compared to some other forms of exercise. | 70 | 30 | 0 | 4  A | 2 | 2  D | 4  A |
| --- | --- | --- | --- | --- | --- | --- | --- | --- | --- | --- | --- | --- | --- | --- | --- | --- | --- |
|  |  |  |  |  |  |  |  |  |  |  | Non-consensus  Study team to make final decision  (see Table S2) | | | | | | |
| **IRTQ50**  Programming | An IRT program takes less time to perform to elicit anti-hypertensive benefits than other types of exercise. | 92.3 | 7.7 | 0.0 | 4  A | 1 | 4  A | 5  SA |  | An IRT program takes less time to perform **and** to elicit anti-hypertensive benefits than other types of exercise. | Included in the final content list after round-1 | | | | | | |

A = agree, D = disagree, DK = don’t know, SA = strongly agree, SD = strongly disagree

1

2

3

4

5

Strongly disagree

Strongly agree

Don’t know

**M**

**IQR**

M = median

IQR = interquartile range of response

Consensus = ≥75%

**Supplementary** **Table 2** Summary of study team’s final decision on remaining non-consensus Round-2 items

| **Item code** | **Description** | **Consensus reached by panellists** | | | **Team’s decision** |
| --- | --- | --- | --- | --- | --- |
|  |  | **Agree** | **Disagree** | **Don’t know** |  |
| **Category: Safety** | | | | | |
| IRT-2 Q1 | Handgrip IRT employed at an appropriate training intensity (e.g. 30% MVC) causes blood pressure responses of >30mmHg in SBP or 20mmHg in DBP. | 40% | 60% | 0% | Not accepted – as more research is needed |
| IRT-2 Q4 | IRT employed at an appropriate training intensity (e.g. 30% MVC for handgrip or 20% MVC for leg) causes increases in heart rate response of >15 beats per minute. | 60% | 40% | 0% | Not accepted – as more research is needed |
| IRT-2 Q7 (reverse of Q1) | Handgrip IRT employed at an appropriate training intensity (e.g. 30% MVC) does not cause blood pressure responses of >30mmHg in SBP or 20mmHg in DBP. | 60% | 30% | 10% | Not accepted – however, likely to occur but not in everyone |
| IRT-2 Q8 | Leg IRT employed at an appropriate training intensity (e.g. 20% MVC) does not cause blood pressure responses of >30mmHg in SBP or 20mmHg in DBP. | 20% | 70% | 10% | Accepted (**disagreement**) |
| **Category: Efficacy** | | | | | |
| IRT-2 Q9 | An appropriate IRT program is beneficial for people with heart disease as it has the potential of eliciting an ischaemic pre-conditioning response. | 70% | 10% | 20% | Accepted (**agreement**) |
| **Category: Programming** | | | | | |
| IRT-2 Q18 | Due to its simplicity a handgrip IRT program prescribed for home-based training will most likely result in higher adherence than an aerobic and/or strength exercise program. | 60% | 20% | 20% | Not accepted – as more research is needed |
| IRT-2 Q19 | Compared to handgrip IRT, a leg IRT program at 20% MVC prescribed for home-based training will most likely result in a higher adherence than an aerobic and/or strength exercise program due to its simplicity. | 20% | 10% | 70% | Not accepted – as more research is needed |
| IRT-2 Q20 | Besides walking, an IRT program is relatively inexpensive compared to some other forms of exercise. | 70% | 30% | 0% | Accepted (**agreement**) |
| **Category: Delivery** | | | | | |
| IRT-2 Q11 | Leg IRT at 20% MVC is simple to deliver as no equipment is required. | 50% | 30% | 20% | Not accepted – as more research is needed |
| IRT-2 Q12 | Leg IRT can be accurately delivered at a precise MVC intensity without the use of gym/laboratory equipment. | 30% | 30% | 40% | Not accepted – as more research is needed |
| IRT-2 Q14 | Leg IRT is more difficult to prescribe as opposed to handgrip IRT as sophisticated laboratory/gym equipment is required to determine the exact MVC necessary to prescribe the appropriate intensity for leg IRT. | 50% | 30% | 20% | Accepted (**agreement**) |
| IRT-2 Q15 | Prescription of handgrip IRT is preferred to leg IRT for home-based delivery as the former is inexpensive and easier to use compared to the later which requires sophisticated laboratory/gym equipment to determine precise MVC. | 50% | 30% | 20% | Accepted (**agreement**) |
| **Category: Mechanism of action** | | | | | |
| IRT-2 Q22 | Repeated exposure of IRT may cause permanent changes in blood vessels (e.g. diameter) with time. Longer term IRT (i.e. ≥6 months) may lead to more consistent blood pressure adaptations. | 70% | 10% | 20% | Accepted (**agreement**) |
| IRT-2 Q23 | Repeated exposure to IRT increases baroreflex sensitivity which may improve cardiac autonomic modulation. | 70% | 0% | 30% | Accepted (**agreement**) |
